# Expert consensus statement for quantitative measurement and morphologic assessment of optical coherence tomography: update 2025

**DOI:** 10.1007/s12928-024-01080-8

**Published:** 2025-01-28

**Authors:** Kenichi Fujii, Takashi Kubo, Hiromasa Otake, Gaku Nakazawa, Shinjo Sonoda, Kiyoshi Hibi, Toshiro Shinke, Yoshio Kobayashi, Yuji Ikari, Ken Kozuma, Takashi Akasaka

**Affiliations:** 1 Division of Cardiology, Department of Medicine II, Kansai Medical University, Hirakata-City, Osaka 5731010 Japan; 2 Division of Cardiology, Hachioji Medical Center, Tokyo Medical University, Hachioji, Japan; 3 Division of Cardiovascular Medicine, Department of Internal Medicine, Kobe University Graduate School of Medicine, Kobe, Japan; 4 Department of Cardiology, Faculty of Medicine, Kindai University, Osaka-Sayama, Japan; 5 Department of Cardiovascular Medicine, Saga University, Saga, Japan; 6 Division of Cardiology, Yokohama City University Medical Center, Yokohama, Japan; 7 Division of Cardiovascular Medicine, Department of Internal Medicine, Showa University School of Medicine, Tokyo, Japan; 8 Department of Cardiovascular Medicine, Chiba University Graduate School of Medicine, Chiba, Japan; 9 Department of Cardiology, Tokai University School of Medicine, Isehara, Japan; 10 Department of Cardiology, Teikyo University School of Medicine, Tokyo, Japan; 11 Department of Cardiovascular Medicine, Nishinomiya Watanabe Cardiovascular Cerebral Center, Nishinomiya, Japan

**Keywords:** Optical coherence tomography, Atherosclerosis, Coronary artery disease

## Abstract

In this updated expert consensus document, the methods for the quantitative measurement and morphologic assessment of optical coherence tomography (OCT) / optical frequency domain imaging images (OFDI) are briefly summarized. The focus is on the clinical application and the clinical evidence of OCT / OFDI to guide percutaneous coronary interventions.

## Introduction

In 2020, the Japanese Association of Cardiovascular Intervention and Therapeutics published an expert consensus document on optical coherence tomography (OCT) / optical frequency domain imaging images (OFDI) [1]. The 2020 consensus document focused solely on standards for the quantitative measurement and morphologic assessment of OCT/OFDI. This document was updated in 2022 to incorporate clinical evidence supporting the use of OCT/OFDI in percutaneous coronary intervention (PCI). Despite the recent European Society of Cardiology guidelines recommending intracoronary imaging guidance with IVUS or OCT when performing PCI on anatomically complex lesions, such as left main stem, true bifurcations, and long lesions [2], the clinical use of OCT/OFDI in PCI still seems to be low worldwide. Therefore, this updated expert consensus document in 2025 provides an additional summary of recent clinical evidence of OCT/OFDI.

OCT was first established for cross-sectional retinal imaging in 1991 by a Massachusetts Institute of Technology team. Then, OCT was first applied in intracoronary imaging in humans and showed promising capability as a tool for diagnostic imaging for arterial wall pathologies in 2001. Since then, OCT/OFDI, a catheter-based imaging modality, has been widely used in the clinical catheterization laboratory for the imaging of the coronary artery wall. With an axial resolution of approximately 10 μm [3], OCT is capable of allowing for the visualization of the coronary artery microstructure at a resolution that is 10-times better than that of intravascular ultrasound (IVUS). Thus, OCT has become one of the commonly used intracoronary imaging techniques during a PCI procedure. OCT provides valuable information that can be used clinically to optimize stent deployment and avoid PCI-related complications [4]. Pre-procedural measurement of lumen and vessel dimensions can facilitate accurate stent sizing [5]. Moreover, pre-procedural assessment of the tissue characteristics of the target lesion can guide optimal treatment strategies. However, OCT has evolved without existing standards for the measurement and the interpretation of images. In this review, we provide a short description of a consistent approach to OCT analysis to assist both clinicians and researchers.

## Quantitative measurements

Measurements should be avoided if motion artifacts are present or incomplete flushing of the intra-coronary blood. In order to provide accurate measurements, the image should be correctly calibrated for z-offset and refractive index. Because coronary artery disease often appears to be more extensive on OCT than on angiography, it is quite difficult to accurately define reference segments in some cases. However, in general, the proximal reference is defined as the site with the largest lumen area, proximal to a stenosis but within the same segment (usually within 10 mm of the stenosis with no major intervening branches). Similarly, the distal reference is the site with the largest lumen area distal to a stenosis but within the same segment (usually within 10 mm of the stenosis with no intervening branches). The reference segment can be used which of these three reference sites (proximal, distal, or average). Quantitative measurements from JACC IVUS Consensus Document have been normally adopted for OCT measurement [6].

### Lumen measurements

Lumen measurements are accomplished using the interface between the lumen and the leading edge of the intima. The following parameters can be measured in each cross-section (mostly automatically):

- Lumen cross-sectional area (CSA): The area bounded by the luminal border.
- Minimum lumen diameter: The shortest diameter through the center of the lumen.
- Maximum lumen diameter: The longest diameter through the center of the lumen. This line does not have to be orthogonal to the line of minimum lumen diameter.
- Lumen Eccentricity: (maximum lumen diameter minus minimum lumen diameter) divided by maximum lumen diameter.
- Percent area stenosis: (Reference lumen CSA minus minimum lumen CSA)/reference lumen CSA. The reference lumen CSA is generally used average lumen CSA of both proximal and distal reference site.

### Vessel (internal elastic membrane) measurements

Vessel and plaque areas are not usually measurable using OCT because OCT has limited depth of penetration, which diminishes the ability to visualize the internal elastic membrane at the cross-section with a large plaque area. For reference segments in which the internal elastic membrane can be identified, can the vessel measurements be made. Because the leading edge of the internal elastic membrane is well delineated on OCT, OCT-based measurements can be used to determine the true histological atheroma area.

### Stent measurements

**Fig. 1:**
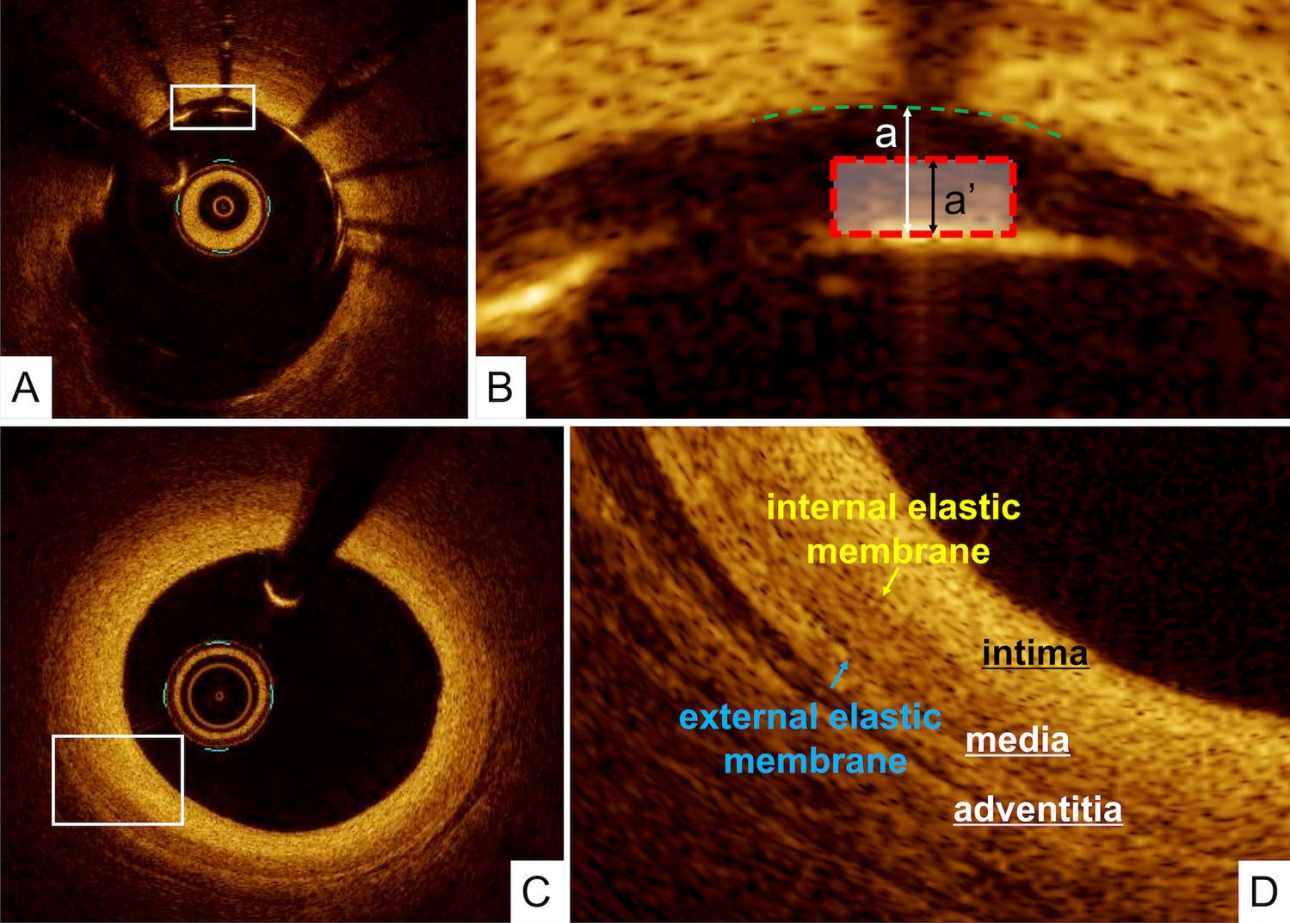
Assessment of strut apposition, and normal coronary artery. **A**, **B** The distance between the abluminal surface of the strut and the estimated vessel wall is measured, extending and joining the contours of the wall on either side of the strut shadow with the measurement line as perpendicular as possible to the strut and vessel wall. Stent malapposition is defined as a distance between the abluminal surface of the strut and the estimated vessel wall (a) greater than the thickness of each stent strut plus polymer (a’). **C**, **D** Normal coronary artery reveals 3 layers, comprising a high-intensity intima, homogeneous low-backscattering media, and heterogeneous high-intensity adventitia. The internal elastic membrane should exit between the intima and media while the external elastic membrane exits between the media and adventitia

Metallic stent struts have a strong reflection coefficient in relation to the light signal used in OCT. Therefore, stents appear as highly reflective surfaces and cast shadows on the vessel wall behind. The following stent-related parameters can be measured using OCT:

- Stent CSA: The area bounded by the luminal border of the stent.
- Minimum stent diameter: The shortest diameter through the center of the stent.
- Maximum stent diameter: The longest diameter through the center of the stent. This line does not have to be orthogonal to the line of minimum stent diameter.
- Stent symmetry: (maximum stent diameter minus minimum stent diameter) divided by maximum stent diameter.
- Percent stent expansion: The minimum stent CSA compared with the predefined average reference area.
- Stent malapposition: When the stent strut is determined to not be fully attached to the vessel wall by visual assessment, the position of a stent strut relative to the vessel wall should be measured by magnifying the individual stent strut to maximize accuracy. Because OCT can show only the luminal surface of the strut owing to the limited depth of penetration through the metal, strut thickness should be considered in evaluating stent apposition for each type of stent design. First, one cursor is positioned on the adluminal surface of the strut, followed by another cursor at the surface of the estimated vessel wall within the stent strut shadow (Fig. 1) [7]. It is important to note that the right position of the adluminal surface of the stent strut falls in the center of the blooming [8]. The measurement line should be as perpendicular to the strut and vessel wall as possible. Stent malapposition can be defined as a distance between the adluminal surface of the strut and the estimated vessel wall greater than the thickness of each stent strut. Some investigators have estimated the location of the adluminal surface of the strut by drawing a line from the surface of the blooming toward the artery wall.
- Neointimal thickness: The distance between the endoluminal surface of the neointima and the luminal surface of the stent strut.
- Uncovered strut: Struts with a measured neointima thickness equal to 0 mm
- Percentage of uncovered strut: The number of struts without distinct overlying tissue, in which the luminal reflection of the strut surface is directly interfacing with the lumen, divided by the total number of analyzed stent struts.

## Qualitative assessment

The normal coronary artery consists of three layers: the intima, consisting of the endothelium and collagen fibers; the media, consisting of smooth muscle cells and elastic fibers; and the adventitia, consisting of collagen fibers and surrounding fat. Because OCT measures the intensity of light returning from a tissue, tissues with a higher heterogeneity in the refractive optical index exhibit stronger optical scattering, and therefore, a stronger OCT signal. The intima in the normal arterial wall is usually represented by the high-intensity signal from the collagen fibers. The media appears as a homogeneous dark layer because of the presence of less collagen fibers and abundant smooth muscle cells and extracellular matrix. The adventitia is also represented by the high-intensity signal from the collagen fibers. (Fig. 1).

### Plaque morphology

**Fig. 2:**
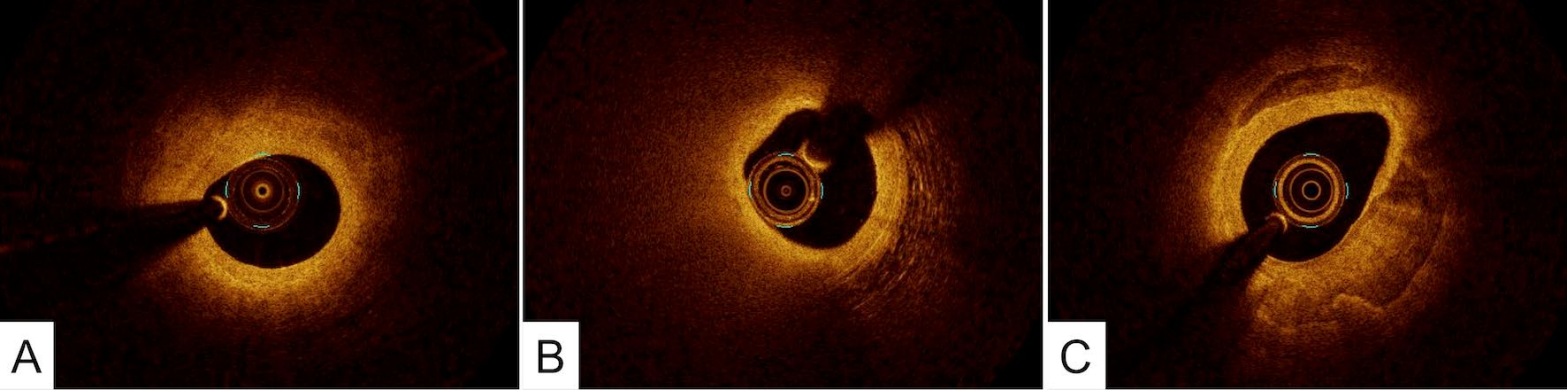
Fibrous, lipid-rich, and calcified plaques. **A** Fibrous plaque appears as a homogeneous high-intensity tissue with a gradual signal attenuation. **B** Lipid-rich plaque appears as low-signal-intensity regions with diffuse borders. **C** Calcified plaque appears as low-signal-intensity areas with sharply delineated borders

OCT is capable of differentiating lipid-rich plaque from fibrous plaque. Because OCT uses near-infrared light and cross-sectional images are generated by measuring the echo-time delay and intensity of light that is reflected or backscattered from the arterial wall, OCT can characterize tissue morphology by measuring the backscattered infrared light. In general, plaques can be characterized as fibrous, lipid-rich, or calcified, according to the plaque characterization criteria developed by histology-validation studies [9–11]. (Fig. 2).

Fibrous plaques: These plaques consist of bundles of collagen fibers, smooth muscle cells, and extra-cellular matrix such as proteoglycan, and appear as a high-signal-intensity tissue because there is much reflected OCT light signal returning from the collagen fibers. Fibrous plaques represent the majority of atherosclerotic lesions. In general, the large amount of collagen fibers does not allow the visualization of the internal and external elastic membrane.

**Fig. 3:**
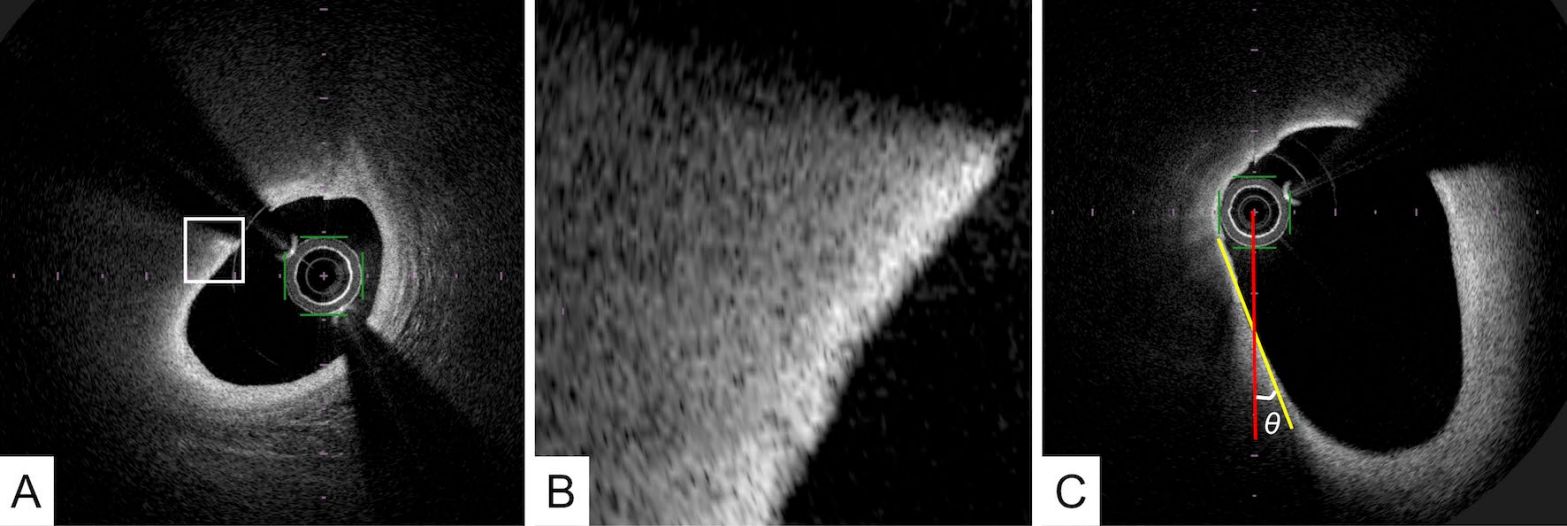
A representative image of thin-cap fibroatheroma. **A** OFDI image indicates signal-poor lesions with an overlying signal-rich band. **B** A magnified image of A. The minimum fibrous cap thickness was 50 µm. **C** A small angle between the line of sight (red line) and the tangent to the lumen contour (yellow line) could lead to a tangential signal dropout

Lipid-rich plaques: These plaques appear as low-signal-intensity regions with diffuse borders because of considerable scattering of light of approximately 1000-nm wavelength from lipid components [12]. Fibroatheroma, which contains a necrotic core, cholesterol crystals, and foam cells, is considered as a representative tissue of a lipid-rich plaque. It should be noted that it is difficult to distinguish between the necrotic core and foam-cell accumulation on a single cross-sectional OCT image because these two tissues have similar OCT attenuation coefficients [13–15]. It has been postulated that thin-cap fibroatheroma (TCFA), which is characterized by a large necrotic core with an overlying thin fibrous cap, measuring < 65 µm, is the precursor plaque composition of plaque ruptures [16]. For OCT analysis, TCFA is usually defined as a plaque with lipid content with a fibrous cap measuring < 65 µm. (Fig. 3) The cap thickness should be measured at its thinnest region within the fibrous cap. However, it should be noted that imaging geometries with a small angle between the line of sight and the tangent to the lumen contour may lead to tangential signal dropout because the imaging beam cannot penetrate the vessel wall owing to strong scattering along an oblique line-of-sight. (Fig. 3C) Therefore, such sections should be interpreted with care.

Calcified plaques: These plaques appear as low-signal-intensity areas with sharply delineated borders. Because the dimensions of the individual particles of calcium hydroxyapatite are smaller than the wavelength of near-infrared light, there is little reflected light returning from these tissues. Thus, OCT images of dense calcium show weaker optical scattering and therefore, a lower OCT signal intensity.

In addition to fibrous, lipid-rich, and calcified plaques, the following morphology has been frequently found in patients with acute coronary syndrome on OCT.

Plaque rupture: A ruptured plaque is defined as a plaque with intimal tearing, disruption, or dissection of the cap. On injection of optically transparent crystalloid or radiocontrast media, these defects may have little or no OCT signal and may appear as cavities.

**Fig. 4:**
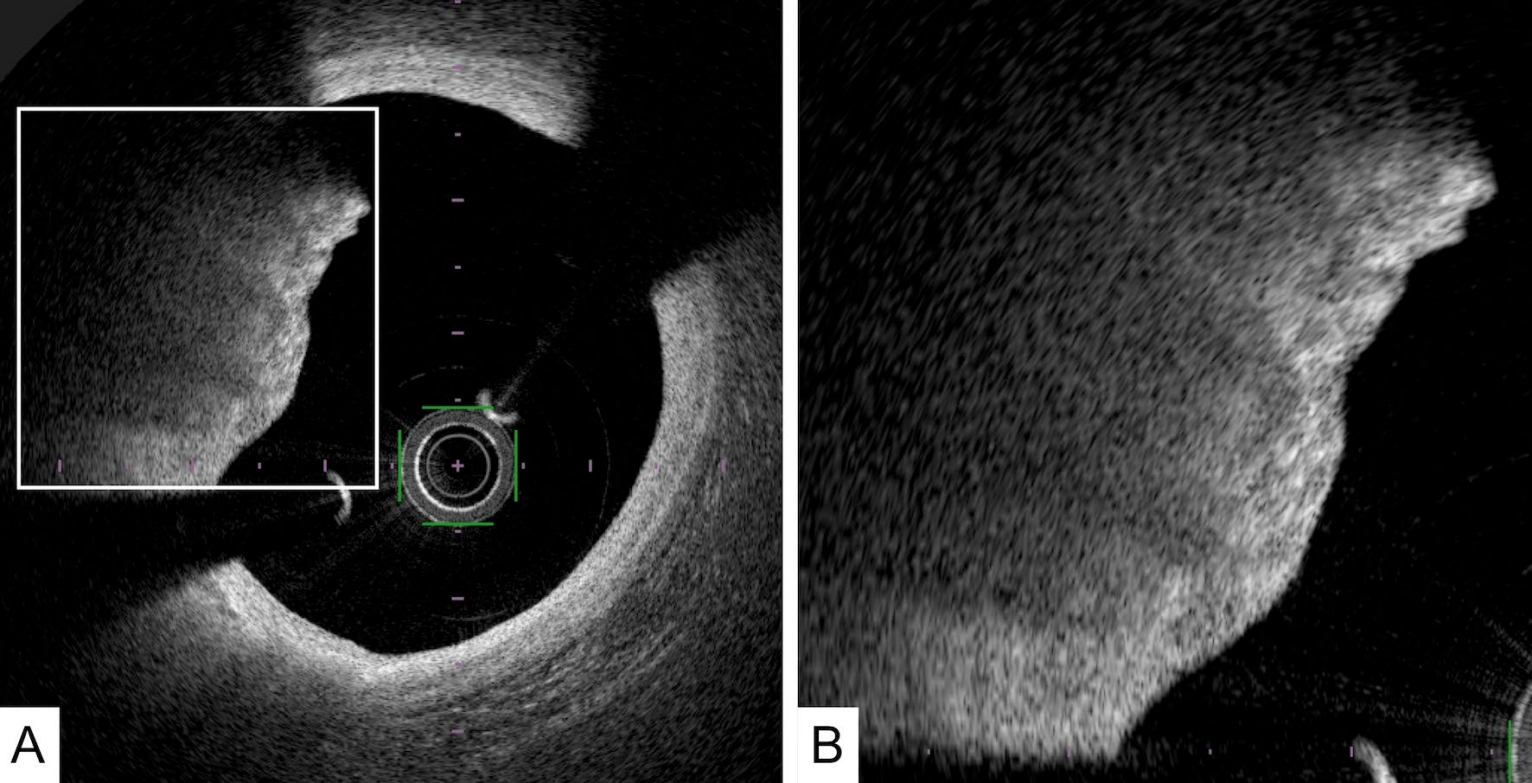
A representative image of a calcified nodule. **A** OFDI image showing a high-backscattering protruding mass with an irregular surface, following a low-intensity area with a diffuse border (arrowheads) such as a red thrombus. **B** A magnified image of A

Erosion: OCT-derived erosion could be composed of intracoronary thrombus attaching to the luminal surface without detectable signs of fibrous cap rupture. However, it should be noted that the resolution of OCT may be insufficient for directly visualizing one layer of endothelial cells in the form of a 5-μm thick cellular monolayer, although OCT has a higher resolution than any other imaging modality,

Calcified nodule: A calcified nodule, which also has the potential to develop into coronary thrombosis, is defined as a high-backscattering mass protruding into the lumen with a strong signal attenuation and an irregular surface [17]. (Fig. 4) The calcified nodule often contains fibrin between the bony spicules, along with osteoblasts, osteoclasts, and inflammatory cells. It should be noted that the OCT image of a calcified nodule is similar to that of a red thrombus, although a calcified nodule usually coexists with a calcified sheet in the same segments.

The other plaque morphologies can be evaluated using OCT.

**Fig. 5:**
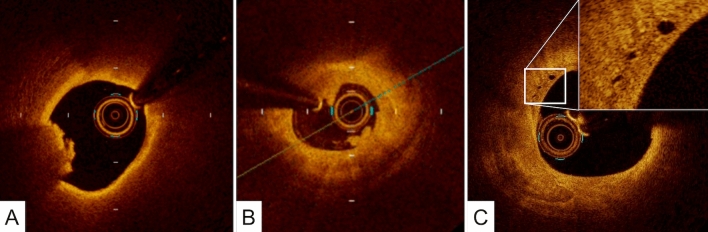
Representative image of a red and white thrombus, and microvessel. **A** Red thrombus appears as an intramural mobile mass protruding into the lumen with a high backscattering and attenuation. **B** White thrombus appears as an intramural mass with homogeneous backscattering and low attenuation. **C** Microvessel appears as a no-signal tubuloluminal structure without a connection to the vessel lumen in multiple contiguous cross-sections

Thrombus: A thrombus appears as an intramural mobile mass attached to the luminal surface or floating within the lumen. OCT can be used to classify a thrombus as a red or white thrombus. A red thrombus is a red blood cell-rich thrombus, which appears as an intramural mobile mass protruding into the lumen with high backscattering and attenuation. (Fig. 5A) A white thrombus is a platelet-rich thrombus that is defined as an intramural mass with homogeneous backscattering and low attenuation. (Fig. 5B) Because red thrombi could be misinterpreted as calcified nodules on OCT, the patient’s background history and lesion morphology (e.g., acute coronary syndrome, on hemodialysis) should be taken into accounts for their interpretation.

Cholesterol crystals: Cholesterol crystals on OCT might appear as a thin, linear region of high signal-intensity within a lipid-rich plaque. However, further histological validation data would be necessary for the accurate detection of cholesterol crystals.

Microvessels: A microvessel appears as a no-signal tubuloluminal structure without a connection to the vessel lumen. These microvessels usually have a sharply delineated border and can be followed in multiple contiguous cross-sections. (Fig. 5C) Owing to the limited penetration depth and axial resolution of OCT, the capability of OCT to reliably detect tiny microvessels in vulnerable atheromatous plaques merits future research. Recanalization of organized thrombus can be identified as multiple large channels, described as having “honeycomb-like appearance”, “swiss cheese appearance”, or “lotus root appearance”.

**Fig. 6:**
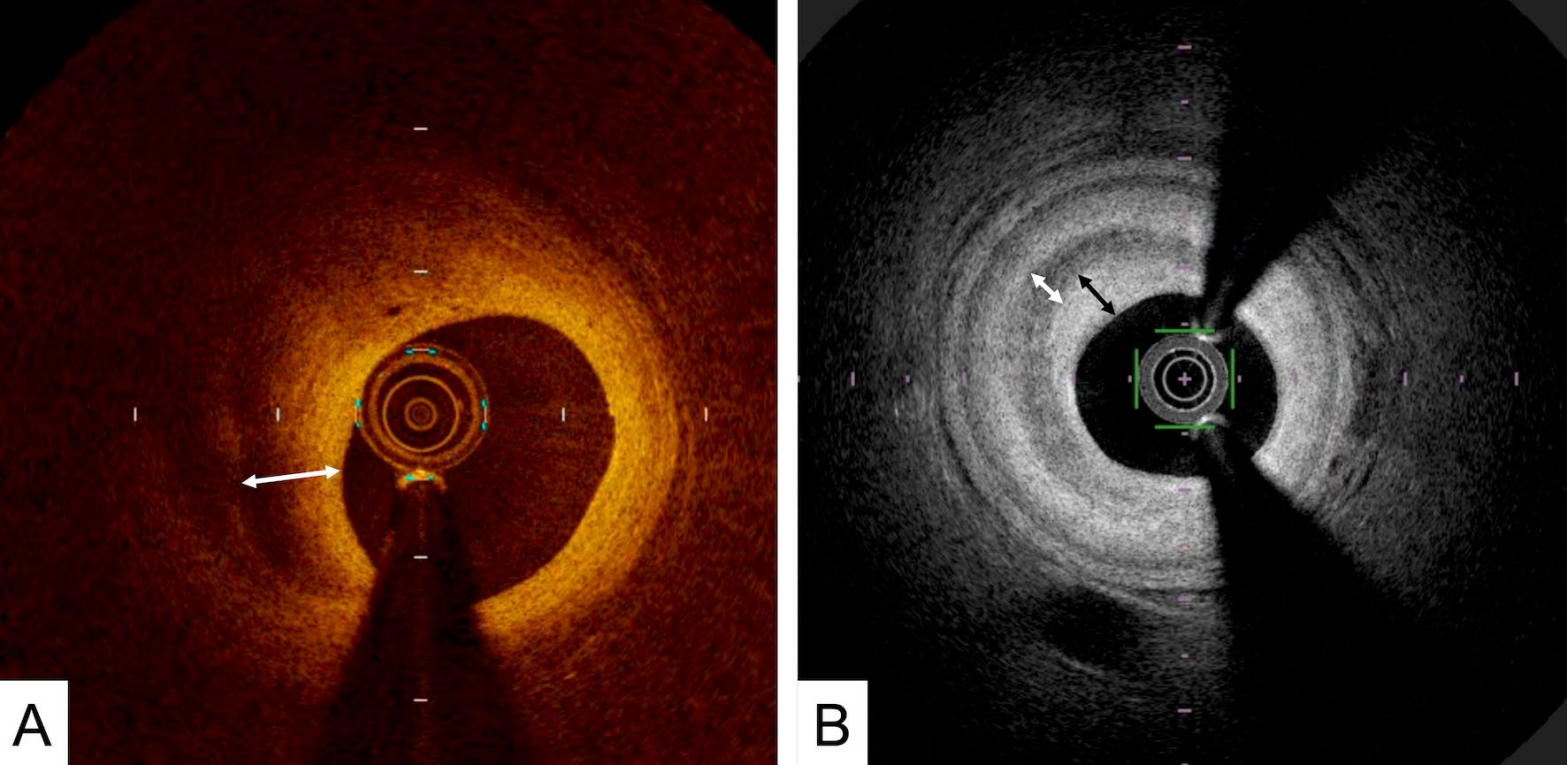
Representative image of a layered plaque. The double-headed arrow indicates a layer with different optical densities. **A** OCT image. **B** In the OFDI, there are three layers of different optical densities

Layered plaque: After plaque rupture or plaque erosion in a coronary artery, a patient does not necessarily develop an acute coronary syndrome. If the degree of stenosis prior to rupture is low, thrombus formation may occur; however, in some cases, the event may not progress [18]. In such instances, the newly formed thrombus organizes and deposits connective tissue, primarily proteoglycans and type III collagen [19, 20], which appears as a band of high backscattered signal on OCT/OFDI. A previous histopathologic validation study reported good agreement between healed plaques observed in pathology and layered plaques seen on OCT [21]. Layered plaque is defined as a plaque with one or more layers of differing optical densities and a clear demarcation from the underlying components (Fig. 6).

Dissection: OCT is commonly employed to detect dissections after interventional stent implantation. The classification of dissections into five categories is recommended:

- Intimal: Limited to the intima or plaque, and not extending to the media.
- Medial: Extending into the media.
- Adventitial: Extending through the external elastic membrane.
- Intramural hematoma: An accumulation of flushing media within the medial space, displacing the internal elastic membrane inward and external elastic membrane outward.
- Intra-stent: Separation of neointimal hyperplasia from stent struts, usually seen only after treatment of in-stent restenosis.

In-stent tissue protrusion: OCT’s high spatial resolution enables the detection and evaluation of tissue protrusions following stent implantation. It is recommended to classify in-stent tissue protrusion into three categories:

- Smooth: Bowing of a plaque into the lumen between the stent struts without intimal disruption.
- Disrupted fibrous tissue: Disruption of the underlying fibrous tissue protrusion into the lumen between the stent struts.
- Irregular: Protruding the material with an irregular surface into the lumen between the stent struts.

## Clinical evidence

The CLI-OPCI trial was the first study to demonstrate the clinical utility of OCT-guidance PCI compared with angiography-guidance PCI [22]. Although this was a retrospective analysis, a total of 670 patients were included, 335 in the OCT-guidance group and 335 in the angiography-guidance group, were included in the study. This study showed that the OCT-guidance group had a significantly lower incidence of one-year cardiac death, cardiac death or myocardial infarction, and the composite of cardiac death, myocardial infarction, or repeat revascularization compared with the angiography-guidance group. Previous studies have revealed a significant clinical benefit of IVUS-guidance PCI during drug-eluting stent implantation over angiography-guidance [23, 24]. Although these trials were performed on relatively simple lesions, two recent randomized trials showed the clinical benefit of OCT/OFDI in PCI for complex lesions. OCTOBER study was a multicenter, randomized, open-label trial, which included patients with a clinical indication for PCI and a complex bifurcation lesion identified by means of coronary angiography were randomly assigned in a 1:1 ratio to OCT-guided PCI or angiography-guided PCI. It showed that OCT-guided PCI in left main and non-left main bifurcation lesions had a lower incidence of a composite of cardiac death, target lesion myocardial infarction, or ischaemia-driven target-lesion revascularization at 2 years compared with angiography only (10.1% versus 14.1%, respectively), and this is despite 15% IVUS use in the angiography arm of whom 64% had a lesion located at a left main coronary-artery bifurcation [25]. The ILUMIEN IV trial, which randomized patients with medically treated diabetes or complex coronary artery lesions to undergo OCT-guided PCI or angiography-guided PCI, showed no benefit in terms of clinical outcomes at 2 years, while the incidence of definite/probable stent thrombosis was significantly reduced with OCT-guided PCI compared to angiography-guided PCI (0.5% vs. 1.4%) [26]. Since OCT has a 10 times higher spatial resolution than IVUS, OCT-guidance PCI may result in superior clinical outcomes compared with IVUS-guidance PCI. The OPINION trial is a multicenter, prospective, randomized, controlled, open-label, parallel group, non-inferiority trial comparing OCT-guidance PCI with IVUS-guidance, which randomized 800 patients 1:1 [4]. In this trial, OFDI-guidance PCI was noninferior to IVUS-guidance PCI for the primary endpoint of target vessel failure at 12 months (5.2% vs. 5.1%). However, in a subgroup analysis, the neointima area tended at 8-month to be smaller in the OFDI-guidance group than in the IVUS-guidance group (0.56 ± 0.30 mm^2^ versus 0.80 ± 0.65 mm^2^, *p* = 0.057). In addition, the percentage of uncovered struts was significantly higher in the OFDI-guidance group than in the IVUS-guidance group (7.0 ± 7.0% versus 4.7 ± 6.4%, *p* = 0.04). In line with the OPINION trial, the ILUMIEN III study revealed the noninferiority of OCT-guidance PCI to IVUS-guidance [27]. Non-inferiority of OFDI-guided PCI to IVUS-guided PCI in patients with acute coronary syndromes was also reported in a multicenter randomized trial conducted in Japan. The OPINION ACS trial was a prospective, multicenter, randomized, open-label, non-inferiority trial comparing OFDI-guided PCI to IVUS-guided PCI in patients with acute coronary syndromes [28]. In this trial, the primary endpoint, the minimum lumen area at 8 months measured by OFDI, was 4.91 mm^2^ in the OFDI-guided PCI group and 4.76 mm^2^ in the IVUS-guided PCI group. This demonstrated the non-inferiority of OFDI guidance to IVUS guidance (1-sided 95% lower CI: − 0.31 mm^2^; *p* < 0.001). In addition, the total tissue protrusion volume was significantly smaller in the OFDI-guided PCI group compared to the IVUS-guided PCI group. Given the numerous demonstrations of the usefulness of OCT/OFDI-guided PCI, the ESC guideline now recommends the use of intracoronary imaging for complex lesions, as mentioned above, with a high level of evidence [2].
